# Low-Dimensional Materials: From Size Confinement to AI-Driven Materials Design Strategy

**DOI:** 10.3390/ma19194145

**Published:** 2026-09-28

**Authors:** Paolo Negro, Federico Cesano

**Affiliations:** Chemistry Department and NIS (Nanomaterial for Industry and Sustainability) Interdepartmental Centre, University of Turin, and INSTM-UdR Torino, Via P. Giuria 7, 10125 Torino, Italy

## 1. Low-Dimensional Materials: When Dimensionality Becomes a Design Variable in Materials Science

Low-dimensional materials are systems in which one or more characteristic dimensions are restricted to the atomic or nanometer scale. They are classified as 0D, 1D, or 2D according to the number of spatial directions where the structure remains extended. This reduced dimensionality can reorganize or quantize electronic and vibrational states, enhance surface and interface effects, and generate properties that are not available in conventional three-dimensional structures. Carbon is a useful example here: atomic carbon arrangements can be found in fullerenes (0D), nanotubes (1D), graphene sheets (2D), and bulk materials, such as graphite and diamond (3D).

The spatial confinement has a physical consequence because charge carriers have wave-like properties. When a material becomes sufficiently small in one or more directions, only specific electronic states are allowed. The continuous energy bands of the bulk material then split into subbands or discrete energy levels, depending on the number of confined directions.

A second consequence can be envisaged due to the fact that low-dimensional materials are increasingly governed by their surfaces, edges, and interfaces. In a macroscopic crystal, most atoms are located in the bulk, but as the thickness or diameter decreases, an increasing proportion of atoms become exposed to the surface. In practical terms, an atomically thin sheet is almost an entire interface. Therefore, adsorbed molecules, neighboring layers, electric fields, strain, and defects can modify a substantial fraction of the material directly. This surface-dominated character is the basis for many applications in sensing, catalysis, and electrochemistry, and it also makes interfaces a powerful tool for controlling electronic, magnetic, and optical responses.

It is also useful to distinguish between structural and electronic dimensionality. For example, a crystal may contain strongly bonded chains or layers within an overall 3D structure, yet its electrons may behave as if confined to one or two dimensions. However, not every nanometer-thick film is necessarily a 2D material. The most significant cases are those in which the interplay of morphology, chemical bonding, and electronic structure results in reduced effective dimensionality. This distinction becomes increasingly important as the field expands beyond naturally layered van der Waals crystals towards non-layered compounds, mixed-dimensional systems, and structures whose low-dimensional character is revealed computationally rather than by simple geometric inspection.

**Figure 1:**
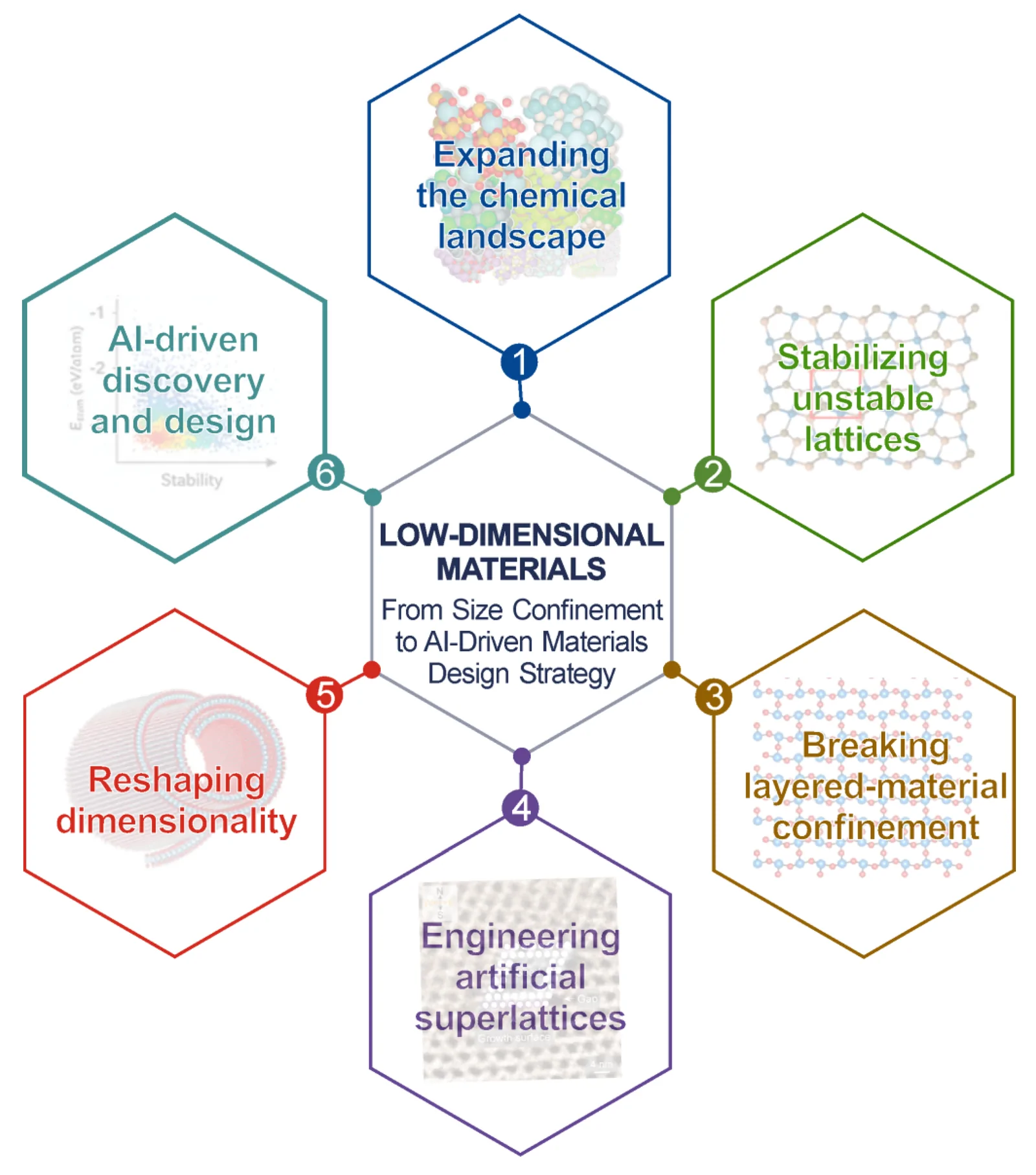
Six emerging directions in low-dimensional materials research: expansion of chemical space, stabilization of metastable lattices, non-layered 2D materials, artificial superlattices, dimensionality conversion, and AI-driven materials design.

As argued in the previous Special Issue, the field of low-dimensional materials has evolved from simply reducing the particle sizes to nanoscale dimensions to increasingly focusing on designing structure, interfaces, dimensionality, and properties [1]. The significance of low dimensionality goes far beyond the original aim of creating thinner models of existing materials. A more ambitious question is emerging: can dimensionality itself be used as a design tool to create phases, lattices, and functions that do not exist in the corresponding bulk material? Recent work suggests that the answer is positive. The six topics discussed below form a logical sequence, ranging from expanding chemistry to stabilizing inaccessible lattices, to the breaking of layered-material confinement, to the engineering of artificial superlattices, to the reshaping of dimensionality post-synthesis, and finally to the use of artificial intelligence (AI) to search for or generate unrealized structures. The six topics are summarized in Figure 1. Together, these six topics represent a transition from the simple isolation of materials to the discovery of new materials.

## 2. Expanding the Chemical Landscape: Exploring 2D Materials Beyond the Known Layered Families

The first frontier in low-dimensional materials is to expand the range of materials from which low-dimensional compounds can be obtained. Traditional mechanical exfoliation naturally favors solids containing weakly bonded layers, whereas chemically derived materials such as MXenes originate from a comparatively restricted set of parent structures. Björk et al. [2] addressed this limitation by combining large-scale computing with selective chemical exfoliation. They screened 66,643 three-dimensional compounds and identified 119 potentially exfoliable candidates distributed across several materials families. Experimental etching of Y from YRu_2_Si_2_ produced flakes of 2D Ru_2_Si_x_O_y_, providing experimental proof of concept for the computational screening strategy. The conceptual advancement is straightforward yet significant: instead of merely considering which crystals can be peeled apart, one can also consider which bulk compounds can be intentionally “broken down” to create a stable, low-dimensional structure. The discovery problem is therefore reframed as designing a transformation pathway through chemical space.

## 3. Stabilizing Unstable Lattices: Metastability as an Opportunity

Once the accessible range of compounds has been broadened, the next challenge is achieving chemical stability through interfacial control. Many theoretically promising 2D lattices are metastable, meaning they correspond to local energy minima and do not necessarily appear as the equilibrium bulk phase. Liu et al. [3] demonstrated that the supporting substrate can act as an active design element by synthesizing a monolayer of pentagonal PdTe_2_ on Pd (100) via symmetry-driven epitaxy. The substrate provides a structural arrangement that favors a low-symmetry pentagonal network, resulting in a semiconducting monolayer with an indirect bandgap of approximately 1.05 eV. The broader lesson is that a substrate is not just an inactive surface on which atoms land. By matching or deliberately inhibiting particular symmetries, a substrate can stabilize atomic arrangements that would otherwise disappear during growth. From this perspective, metastability is not just a synthetic obstacle, but a source of potentially useful structures that can be accessed through interfacial control.

## 4. Breaking the Barrier of Layered Materials: Atomically Thin Crystals Derived from Non-Layered Solids

An alternative frontier strategy is to remove the idea that the parent solid must be layered. This statement is important because most known inorganic compounds are not made up of weakly bonded sheets and therefore cannot be isolated by conventional exfoliation methods. This is also relevant from a technological perspective in the context of 2D electronics, where the limited availability of high-performance p-type semiconductors makes it difficult to implement complementary logic. Xiong et al. [4] reported the growth of ultrathin, non-layered β-Bi_2_O_3_ crystals on a SiO_2_/Si substrate through a vapor–liquid–solid–solid route involving a layered BiOCl intermediate. The resulting 2D β-Bi_2_O_3_ crystals were thinner than 1 nm and supported p-type field-effect transistors with a room-temperature hole mobility of 136.6 cm^2^ V^−1^ s^−1^ and an on/off current ratio of approximately 1.2 × 10^8^. The key idea is that a material does not need to possess a natural van der Waals interaction to reach the 2D structure, as an appropriately engineered kinetic pathway can pass temporarily through a layered intermediate and enable an ultrathin two-dimensional form of a normally three-dimensional compound.

## 5. Designing Artificial Superlattices: From Stacking to Bottom-Up Structural Information

The design space of low-dimensional structures further expands when structural periodicity itself becomes programmable. In atomic van der Waals heterostructures, a small twist angle or lattice-constant mismatch between two periodic layers produces a long-period moiré superlattice. The resulting spatial modulation of local stacking registry can generate a periodic electronic potential and reconstruct the band structure. Twist angle therefore becomes a materials-design variable alongside composition, thickness, strain, and stacking sequence. Such structures are often fabricated by transferring and rotationally aligning atomically thin crystals, a process that requires stringent control over angular alignment, layer registry, and interfacial cleanliness.

Jing et al. [5] extended moiré engineering to the intermediate nanometer scale using bottom-up DNA nanotechnology. Twisted DNA-origami nanoseeds directed the nucleation and growth of stacked two-dimensional single-stranded-tile sublattices with prescribed twist angles, interlayer spacings, stacking sequences, and square, kagome, or honeycomb symmetries. The approach produced sublattice constants as small as approximately 2 nm, moiré periodicities spanning tens of nanometers, and bilayer fractions of up to 90%. The authors also demonstrated a gradient moiré superlattice in which the periodicity varied spatially across the structure.

These architectures bridge the structural gap between angstrom-scale van der Waals moirés and submicrometric-scale photonic systems and have no natural counterparts. The conceptual advancement is therefore from stacking existing sheets to encoding the structural information that determines how an artificial superlattice assembles. This is a particularly clear example of matter being designed through dimensionality rather than merely being miniaturized.

## 6. Dimensionality Conversion: Converting 2D Structures into 1D Functional Architectures

Another direction for research is to challenge the assumption that the dimensionality of a material is fixed once it has been synthesized. Zhang et al. [6] reported a scalable method of converting different MXene compositions (i.e., Ti_2_CT_x_, Ti_3_C_2_T_x_, Ti_3_CNT_x_, V_2_CT_x_, Nb_2_CT_x_, and Ta_4_C_3_T_x_) into one-dimensional scrolls, with production demonstrated at the gram scale. A scroll can be imagined as an atomically thin sheet curling repeatedly around itself to produce an open-ended, tubular architecture. This change in structure is not just surface-level: the curvature, interlayer spacing, and newly created transport pathways can modify the movement of electrons, ions, and molecules through the material. In Nb_2_CT_x_ (T_x_ = oxygen, sulfur, or selenium), scrolled films exhibited approximately 33 times the electrical conductivity of flake-based films and entered a superconducting state below 5.2 K. Additionally, scroll assemblies displayed lower density, enhanced mass transport, and improved performance in supercapacitor electrodes and humidity sensors. This 2D-to-1D conversion illustrates a broader principle. A low-dimensional sheet can become a building block in its own right, whose curvature, topology, and hierarchical assembly create functions absent from the parent flake. In other words, dimensionality does not have to be an outcome of synthesis alone because it can also be modified afterwards. Such an approach challenges the traditional boundaries between 0D, 1D, and 2D classes, encouraging a more functional perspective that considers which confinement geometry best controls transport, the accessibility of active sites, and the coupling between neighboring structural units.

## 7. AI-Driven Discovery and Inverse Materials Design: From Hidden Structure Identification to New Structure Generation

The sixth direction offers a computational approach to complement these experimental advances. Traditional materials discovery typically starts with a known or hypothesized structure and considers the properties it might possess. High-throughput computation has expanded this logic by screening thousands of candidates. However, the underlying calculations can still be costly, and conventional geometric descriptors may overlook unusual low-dimensional motifs. Bagheri et al. [7] combined universal machine learning interatomic potentials with a force-constant-based dimensionality classifier to identify structures whose low-dimensional character is encoded in chemical bonding rather than obvious geometry. After benchmarking the machine learning models against first-principles phonon and force-constant data, the authors screened over 150,000 bulk structures and identified 9139 low-dimensional materials, including 3057 two-dimensional (2D) systems. Of these, 887 were classified as easily or potentially exfoliable and were absent from reference 2D material databases. Here, AI is used not simply to predict one property but to change the scale and even the definition of the search itself. This work is part of a rapid development from prediction to inverse design of materials. In 2023, Merchant et al. [8] introduced Graph Networks for Materials Exploration (GNoME), a graph-neural-network-based approach for screening very large sets of candidate inorganic crystal structures. Selected candidates were then evaluated using density functional theory (DFT), and the resulting calculations were fed back into the model through an iterative active-learning cycle.

In 2025, Zeni et al. [9] introduced MatterGen, a diffusion-based generative model that extends materials discovery from screening to inverse design. Instead of selecting candidates from a predefined set, MatterGen generates new crystal structures by progressively refining atomic species, positions, and lattice parameters, and can be guided towards specified chemical compositions, crystal symmetries, or target mechanical, electronic, and magnetic properties. Generated candidates were subsequently evaluated using DFT calculations to assess their stability and target properties. As a proof of concept, one MatterGen-designed material was also successfully synthesized and experimentally characterized. Okabe et al. [10] introduced Structural Constraint Integration in a GENerative model (SCIGEN), a framework that extends generative materials design by incorporating explicit geometrical constraints into the generation process. SCIGEN guides the generation process towards structures containing prescribed lattice geometries, including honeycomb and kagome motifs and other Archimedean and Lieb-type lattices. Approximately ten million candidate structures were generated, of which more than 10% passed multistage stability screening; around 26,000 selected candidates were subsequently evaluated by high-throughput DFT calculations. Two predicted materials were also experimentally synthesized and characterized.

The design space is exceptionally large for low-dimensional systems because the composition is not the only variable. The function can be altered by thickness, defect distribution, edge structure, strain, stacking order, twist angle, curvature and interfacial registry. While AI can help navigate this combinatorial landscape, prediction does not eliminate the need for physics or experimentation. Candidate structures still require validation based on first principles, assessment of thermodynamic and dynamic stability, identification of realistic synthesis pathways, and ultimately, experimental validation. Therefore, the most credible future is not one where autonomous AI replaces materials science, but rather a closed discovery loop in which machine learning proposes, physics filters, experiments test, and the resulting data improves the next generation of models.

## 8. The Special Issue in Perspective: Seven Contributions Across Structure, Processing and Function

The contributions collected in the Special Issue, “Low-Dimensional Structures for Smart Materials and Composites: Preparation, Properties and Applications (2nd edition)” offer complementary perspectives on the field. Rather than focusing on a single material family, the contributions span theoretical mechanics, surface nanostructuring, graphene processing and stability, zero-dimensional semiconductor and polymer nanoparticles, photovoltaics, sensing, and machine learning-assisted modeling. Taken together, these contributions demonstrate how low dimensionality operates at different levels: as a geometric constraint, a means of controlling surfaces and interfaces, and a parameter for tuning mechanical, transport, and optical responses. They also show how low-dimensional systems are increasingly becoming data-rich platforms whose behavior can be modeled and predicted computationally.

### 8.1. Mechanics and Dimensionality: Predicting the Response of Emerging 1D and 2D Structures

Fernandes et al. [11] address a fundamental prerequisite for the development of devices from theoretically predicted low-dimensional compounds: their mechanical reliability. Using a nanoscale continuum and finite-element analysis, they evaluate the surface Young’s modulus, shear modulus, and Poisson’s ratio of GeC and SnC nanosheets, as well as the corresponding single-walled nanotubes. This study is particularly relevant given that germanium carbide and tin carbide remain largely theoretical despite their proposed roles in nanoelectronics, photocatalysis, batteries and fuel cells. By comparing one-dimensional nanotubes, two-dimensional sheets and their three-dimensional counterparts, the work clarifies how dimensional reduction affects the elastic response and provides mechanical parameters relevant to the design of future low-dimensional devices. Notably, the authors also present a robust method for estimating nanotube elastic constants that does not require the repetition of full numerical simulations. This provides a transferable design tool for the future modeling of low-dimensional carbides that are currently under-explored.

### 8.2. Surface and Interface Engineering: Morphology, Wettability and Device Reproducibility

Corsaro et al. [12] show how micro- and nanoscale surface structuring can impart controlled interfacial functionality to bulk materials. Picosecond laser surface texturing generates periodic microchannels decorated with laser-induced periodic surface structures (LIPSS) on various metals. The morphology is mainly controlled by the parameters of the laser and the scan head. The resulting water contact angles range from approximately 20° to 100°, depending on the material and treatment, demonstrating tunable hydrophilic-to-hydrophobic behavior. A key finding is that wettability cannot be explained by roughness alone. Surface chemistry, oxidation, adsorption of airborne species, and aging all contribute to the time-dependent transition. The study therefore links nanoscale surface patterning to a practical issue in coating and interface technologies where controlled wetting is necessary for adhesion, conformal growth, and reliable processing.

Shmidt et al. [13] investigate how conventional device fabrication affects the structural and electronic quality of graphene. Graphene chips intended for biosensing applications were examined using atomic force microscopy, Raman spectroscopy, and low-frequency noise measurements. Residues originating from photoresist processing caused local contamination and increased RMS roughness by up to an order of magnitude, resulting in a broader range of chip resistance values (approximately 1–10 kΩ). This directly compromised device-to-device reproducibility. Introducing AFM-guided control and additional post-lithography cleaning reduced the resistance spread to approximately 1–1.6 kΩ and restored the roughness to a level similar to that of the initial graphene. Low-frequency noise also proved to be an effective tool for probing defects and inhomogeneous compressive stress. This study highlights a key lesson for 2D technologies: device performance depends not only on the intrinsic properties of graphene, but also on maintaining an atomically clean and mechanically uniform interface throughout the fabrication process.

### 8.3. Nanocrystal Engineering for Photovoltaic Interfaces: From Growth Control to Functional Oxide Composites

In a first contribution, Hattori et al. [14] present a solution-based approach to controlling zero-dimensional SnO_2_ nanocrystals, which are then assembled into thin electron-transport layers for use in perovskite solar cells. Phenylalanine methyl ester hydrochloride acts as a structure-directing and stabilizing ligand, enabling the formation of crystalline SnO_2_ particles that are only a few nanometers in size and have a narrow size distribution, without the need for a catalyst. The reaction temperature provides control over particle growth, dispersion, and self-assembly: well-dispersed particles measuring approximately 2.4 nm are obtained at around 70 °C, while slightly larger particles and increased assembly occur at around 80 °C. The resulting nanofluids can be dispersed in ethanol and deposited by spin coating, thus linking molecular-level synthesis to scalable film formation. This study is significant because the connectivity, dispersion, and surface chemistry of the nanostructured electron-transport layer are ultimately determined by the size and organization of its nanoscale building blocks.

In a second contribution, Hattori et al. [15] extend the scope of this nanocrystal platform by including compositional and electronic engineering, as well as morphological control. A precursor containing lithium chloride and titanium isopropoxide is deposited on the SnO_2_ nanoaggregate layer and thermally treated to produce simultaneous Li incorporation and the formation of an SnO_2_-based solid solution containing a small TiO_2_ fraction. X-ray diffraction and high-resolution TEM analysis confirm the structural evolution of the mixed oxide, and the estimated optical bandgap of approximately 3.45 eV is narrower than that of pristine SnO_2_, consistent with modification of the electronic structure. This approach therefore combines several levels of control, including nanoparticle synthesis, nanoscale interconnection, dopant incorporation, and control of thin-film composition, within a single electron-transport architecture. In the context of perovskite photovoltaics, this demonstrates how low-dimensional building blocks can be chemically reconfigured after assembly to optimize charge extraction and transport, rather than simply acting as passive nanoparticles.

### 8.4. Sensing, Photophysics and Data-Driven Functionality in 0D Nanostructures

Li et al. [16] exploit the large accessible surface area and confined morphology of polymer nanoparticles to enable the direct fluorescence detection of 2,4,6-trinitrophenol (TNP) in water. Cross-linked poly(tetraphenylethene-co-biphenyl) nanoparticles were synthesized using Suzuki miniemulsion polymerization to form porous spheres measuring approximately 56 nm in diameter, with a specific surface area of 69 m^2^/g and pores centered near 2.5 nm. The incorporation of tetraphenylethene suppresses aggregation-caused fluorescence quenching and enables appreciable fluorescence in aqueous nanoparticle dispersions. The system achieves a Stern–Volmer quenching constant of 2.50 × 10^4^ M^−1^ and a TNP detection limit of 1.07 μM, with minimal interference from other nitroaromatic compounds. Tests in spiked environmental water samples reinforce the practical relevance of this method. This study demonstrates how the morphology, porosity, and molecular photophysics of 0D structures can be designed together to convert a nanoscale structure directly into selective chemical sensing.

Malashin et al. [17] introduce a data-driven dimension to the Special Issue by coupling the photophysics of colloidal CdS quantum dots with long short-term memory (LSTM) neural networks. Temperature alters exciton–phonon interactions, recombination pathways, and photoluminescence intensity in quantum-confined nanocrystals, producing complex temperature-dependent trends that can be difficult to describe accurately using simple empirical models. The authors trained an LSTM model using experimental time-series data that linked temperature with integrated photoluminescence intensity, and they used particle-swarm optimization to select effective network hyperparameters. The resulting model reproduces the main temperature-dependent PL trends and shows that machine learning can complement mechanistic spectroscopy when large sequential datasets are available. Unlike AI-driven crystal discovery, this contribution uses AI to predict the behavior of an experimentally characterized 0D material, providing a useful bridge between low-dimensional photophysics and sensing, and the broader move towards data-assisted materials characterization.

## 9. Outlook and Conclusions

Taken together, these seven papers expand the editorial perspective. The theoretical prediction of mechanical response determines the physical viability of new 1D and 2D structures, while surface texturing and graphene process control demonstrates that function is often determined at interfaces. The two SnO_2_ studies connect nanoscale synthesis with charge-transport architectures, polymer nanoparticles translate porosity and confinement into selective sensing, and the CdS study shows how machine learning can extract predictive value from the complex response of quantum-confined systems. Thus, the Special Issue captures low dimensionality not as a single material class but as a cross-cutting strategy for controlling mechanics, interfaces, transport, optical response, and information.
